# Exploring the Impact of a Low-Dose Mindfulness Intervention on Perceived Exertion, Attention, and Rowing Performance

**DOI:** 10.3390/jfmk10040465

**Published:** 2025-12-01

**Authors:** Rebekah Bakken-Gay, Sarah E. Junkersfeld, Robyn Braun-Trocchio

**Affiliations:** Department of Kinesiology, Texas Christian University, Fort Worth, TX 76129, USA; s.brown13@tcu.edu

**Keywords:** Headspace, physical activity, exercise psychology, mindfulness-based intervention, exercisers

## Abstract

**Objectives**: This study examined the effects of a four-week low-dose mindfulness intervention on ratings of perceived exertion, heart rate, attentional allocation, and performance outcomes, including power output, distance rowed, and strokes per minute, during a rowing task. **Methods**: Thirty-two participants between 18 and 37 years of age (21.09 ± 3.67) who met the World Health Organization (WHO) physical activity guidelines and had no previous experience with mindfulness or meditation completed a four-week intervention. Participants were either in the mindfulness intervention (*n* = 17) or the placebo group (*n* = 15). Participants completed ten visits over four weeks, each consisting of watching an episode of either *Headspace Guide to Meditation* or *Wild Babies* on Netflix, followed by a 25 min rowing task. **Results**: Results indicated no significant group-by-session interaction for any variables. However, a significant main effect for session showed that perceived exertion was significantly lower at the post-assessment compared to the pre-assessment for all participants (*p* = 0.013, *η_p_*^2^ = 0.19). Additionally, a significant main effect for time revealed a linear increase in perceived exertion across the rowing task (*p* < 0.001, *η_p_*^2^ = 0.81). Both groups also showed a significant shift from dissociative to associative attention (*p* < 0.001, *η_p_*^2^ = 0.25). For performance, a significant main effect for session was observed, with greater power output (*p* = 0.008, *η_p_*^2^ = 0.22) and distance rowed (*p* = 0.013, *η_p_*^2^ = 0.19) at the post-assessment for both groups. **Conclusions**: The lack of significant group differences suggests that a low-dose, pre-exercise mindfulness video intervention is likely ineffective for altering psychophysiological responses, indicating that future research should prioritize higher-dosage or real-time guided interventions.

## 1. Introduction

Mindfulness has emerged as a buzzword across various contexts, including healthcare and self-care, yet its meaning is often inadequately defined by many who use it [1]. Mindfulness, defined as “paying attention in a particular way: on purpose, in the present moment, and nonjudgmentally,” is a concept that can pose challenges even for the most experienced practitioners [2] (p. 4). While awareness of the present and acceptance of the moment are key components of being mindful, many individuals struggle to understand what it means to accept their thoughts [3]. To practice mindfulness, an individual must override automatic thoughts and the habitual assignment of directionality to their thinking. This shift is thought to allow for active engagement in life, rather than a passive drift through daily experiences. This can be accomplished through mindful meditation, first focusing on breathing and developing neutral thoughts, and then gradually expanding it to other aspects of one’s life, like walking, eating, and even washing dishes [4]. By taking time to be mindfully aware and present, it forces an individual to take an active role in their thoughts and be purposeful in accepting all thoughts.

The roots of mindfulness can be traced back to traditional Buddhist practice, but the mindfulness that is practiced today has evolved. Within sport and exercise psychology, mindfulness-based interventions have been widely investigated. Comprehensive analyses indicate that such programs yield small to moderate positive effects on athletic performance, anxiety reduction, and psychological well-being [5,6]. Beyond the sport context, mindfulness-based interventions have been shown to increase physical activity, enhance self-regulation, and improve body awareness [7,8] and reductions in depression, anxiety, and stress, along with improvements in well-being [9].

The three primary mindfulness interventions used in applied sport and exercise contexts are (1) the Mindful-Acceptance Commitment (MAC), (2) Mindfulness Meditation Training in Sport (MMTS), and (3) Mindful Sport Performance Enhancement (MSPE) [10]. These multi-week, high-contact programs rely on trained facilitators and structured instruction, demanding substantial participant time. While effective, such intensive protocols can limit accessibility and adherence, particularly for exercisers without access to trained practitioners [11].

In contrast, the current study utilizes a low-dose, passive, and technology-delivered format. Platforms like Netflix, Calm, and Headspace provide brief, user-friendly content that the general population can access without formal instruction [12]. The Netflix docuseries *Headspace Guide to Meditation* is an example of how the content from the Headspace app can be redesigned into longer sessions and still make an impact on users [13]. The first ten minutes of each episode contain teaching aspects of what mindfulness is and how to apply it in each of the scenarios, and then the final ten minutes are a guided mindfulness meditation [11,13].

This low-intensity, media-delivered format reflects how most people engage with mindfulness, informally, briefly, and on-demand, rather than through structured, facilitator-led programs. Its selection allows researchers to investigate the minimum effective dose of mindfulness in an ecologically valid, low-cost medium. Despite their widespread use, there is a scarcity of research investigating the effects of those brief, technology-based interventions on psychophysiological responses during exercise [12,14]. Specifically, there is a call for research into more accessible, technology-delivered formats of interventions [15]. By using this Netflix series, the study evaluates whether foundational mindfulness instruction, delivered passively and with minimal time investment, can meaningfully influence exercise-related perceptual and physiological responses, including ratings of perceived exertion (RPE) and heart rate (HR).

Mindfulness interventions emphasize cultivating awareness and acceptance, and these principles can also extend to physical domains like exercise, where perceived exertion and attentional allocation critically influence physical activity behaviors [16]. Once an individual has taken an active role in their consciousness by utilizing mindfulness, there may be a manipulation of their perception of exertion [17]. RPE, a psychophysiological measure, is used to determine the subjective intensity of effort, strain, discomfort, and/or fatigue, and was coined by Gunnar Borg [18] to measure perceptual intensity during exercise. The Borg 6–20 RPE Scale and HR are highly correlated, meaning that one can accurately predict the other, although this prediction’s accuracy depends on individual factors such as age, sex, and fitness level [18,19].

Given the established relationship between perceived exertion and physiological responses, recent research has explored how mindfulness can influence RPE and enhance the overall exercise experience. This includes assisting individuals in detaching from sensory pain during exercise and improving their exercise experience [20]. This aligns with research on mindful cycling, which suggests that mindfulness improves self-awareness of physiological cues, such as HR, and facilitates more accurate predictions of perceived exertion levels that correspond with actual HR experienced during physical activity [17]. Both studies conclude that engaging in mindful exercise enhances the enjoyment and long-term sustainability of physical activity by helping individuals block or reframe the typical negative sensations associated with such activities [17,20]. However, the literature presents conflicting findings on the relationship between mindfulness and RPE, with one study showing that mindful cyclists more accurately aligned RPE with HR [17]. Another found that mindful exercisers reported lower RPE during a walking task compared to a placebo group [21]. More recently, mindful walkers reported lower average RPE before and after the task compared to walkers with no stimulation [22]. Evaluating subjective measures such as RPE presents inherent challenges due to inter-individual variability in perception and psychological state. This creates a need for further investigation into the impact of mindful exercise on RPE to fully understand the psychophysiological relationship between these constructs.

Mindfulness not only impacts the physical sensations while exercising, but also the focus of attention [23]. Attention can be classified as either associative or dissociative [24]. Associative attention allocation involves focusing on the body and internal sensations, while dissociative attention allocation refers to directing focus away from the body, engaging with external stimuli, and deliberately avoiding attention to bodily sensations [24]. Mindfulness is not definitively associative attention, but it shares similar properties that emphasize self-awareness and attention to bodily sensations seen in associative attention [23]. While associative attention may heighten sensitivity to physiological symptoms, mindfulness facilitates the reappraisal of these sensations through nonjudgmental acceptance. This distinction fosters a more adaptive approach to physical activity challenges, allowing individuals to observe discomfort without reacting negatively to it [23,25]. The nonjudgmental aspect of mindfulness is what differentiates it from associative attention. For example, an associative thought could be “my arms are burning, I can’t continue”, whereas a mindful thought would note that there is heat or tension in the biceps, and the individual would move forward without letting the mind dwell on the sensation.

Those who are mindful and maintain heightened awareness of their present experience may perceive tasks to be longer but also tend to enjoy those tasks to a greater degree than those not exhibiting mindfulness [4]. This can be applied to physical activity, where an individual may perceive their exercise bout to be longer, but once it is over, they have a higher level of enjoyment than if they completed the physical activity without being mindful. It has been suggested that an associative strategy that directs attention toward painful sensations during exercise may enhance performance by facilitating effort regulation [26]. Similarly, research on mindfulness during physical activity found that the associative state was associated with greater enjoyment, remembered affect, and forecasted affect compared to a dissociative state induced by music [27]. However, the majority of current literature proposes the use of dissociative attentional strategies to perform exercise tasks and improve performance [28,29,30], highlighting the novelty and innovation of mindfulness-based physical activity interventions that seemingly contradict what has long been emphasized as the standard.

The purpose of the current study was to examine the effects of a four-week low-dose mindfulness intervention on RPE, HR, attentional allocation, and performance outcomes, including power output, distance rowed, and strokes per minute, during a rowing task. This study was guided by two main research questions: (1) How will a four-week low-dose mindfulness intervention impact RPE, HR, and attentional allocation during a rowing task, and (2) How will a four-week low-dose mindfulness intervention impact performance outcome variables of power output, distance rowed, and strokes per minute. It was hypothesized that participants in the intervention group would demonstrate lower RPE, potentially leading to higher HR due to a reduced perception of effort, allowing for greater work output, and a greater shift toward associative attention compared to those in the placebo group during a rowing task. It was also hypothesized that the intervention group would demonstrate greater power output, distance rowed, and strokes per minute compared to the placebo group.

## 2. Materials and Methods

### 2.1. Participants

To determine sample size, G*Power version 3.1.9.7 [31] was used to conduct an a priori repeated measures within-between analysis of variance (RM-ANOVA) and determined the number of participants required for the study was 28, *f* = 0.25, based on convention for a medium effect size in similar psychological interventions [21], α = 0.05, (1 − β) = 0.80, for a design with two groups and three measurements. Thus, the obtained sample size of *n* = 32 was adequate to test the study hypotheses. The total of 32 participants (*n*_female_ = 25, *n*_male_ = 7) between the ages of 18 and 37 years of age (21.09 ± 3.67) completed the intervention in totality of all ten visits. Participants were randomly assigned, using a computer-generated random number sequence, to either the mindfulness intervention (*n* = 17) or the placebo group (*n* = 15). Majority of participants identified as White (*n* = 29) and not Hispanic or Latinx (*n* = 25). The primary occupation of participants was student (*n* = 29), followed by professional (*n* = 3). The inclusion criterion was that individuals met current physical activity guidelines of 150 moderate or 75 vigorous minutes of exercise per week. Exclusion criteria included currently practicing mindfulness or meditation, and a diagnosis of attention-deficit hyperactivity disorder (ADHD) due to potential confounds related to attentional focus during the intervention and task.

### 2.2. Instrumentation

Demographic Questionnaire. A demographic questionnaire was administered to collect information about the participant’s age, sex, race, ethnicity, exercise history, any diagnosis of ADHD, as well as history with mindfulness and meditation. Individuals were screened to assess whether they had practiced yoga, meditation, or mindfulness in the last six months or longer, and were excluded from participation if they had.
  International Physical Activity Questionnaire (IPAQ). The International Physical Activity Questionnaire (IPAQ) short form was used to determine if participants met the inclusion criteria of 150 min of moderate or 75 min of vigorous aerobic physical activity each week [32]. The questionnaire consists of a 7-day physical activity recall, asking about both moderate and vigorous intensity physical activity.
Rating Perceived Exertion (RPE). The Borg RPE 15-point category-ratio scale, ranging from 6 (no exertion at all) to 20 (maximal exertion), was used to measure perceived exertion [18,19]. The higher the RPE score, the higher the perceived exertion.
Attention Scale. Attentional focus was measured using the 10-point scale with “0” meaning external thoughts (*daydreaming, environment, singing songs*) and “10” being internal thoughts (*how the body feels, breathing, muscles*) [33]. Lower scores represent more dissociative attention, with higher scores indicating more associative attention.
Heart Rate (HR). HR was obtained utilizing a Polar H10 sensor with a Pro Strap that sent data instantly to the Polar Beat app via an iPad (Polar Electro Oy, Kempele, Finland).
Performance Outcome Variables. Performance outcome variables of power output (watts), distance rowed (meters), and strokes per minute were recorded from the Aviron rower (Aviron Interactive Inc., Toronto, ON, Canada).
Netflix Series. Each digital mindfulness group visit consisted of watching a Netflix docuseries episode, *Headspace Guide to Meditation* [13]. Narrated by the founder of the app Headspace, Andy Puddicombe, each episode guides the viewer through mindfulness education and guided practice, with accompanying images to sustain attention. The series consists of eight episodes, with titles of (1) “How to Get Started,” (2) “How to Let Go,” (3) “How to Fall in Love with Life,” (4) “How to Deal with Stress,” (5) “How to be Kind,” (6) “How to Deal with Pain,” (7) “How to Deal with Anger,” and (8) “How to Achieve Your Limitless Potential.” The use of this series as a low-dose mindfulness intervention, as previously used in research [11], as a low-cost, readily accessible intervention to explore the transferable effects of teachings before exercise, as compared to previous research that has only investigated a guided meditation delivered during the exercise task [22,27].

Each placebo group visit consisted of watching a Netflix docuseries *Wild Babies* episode, a nature series about the lives of baby animals narrated by Helena Bonham Carter [34]. The series consists of eight episodes, with titles of (1) “New Arrivals,” (2) “Home Alone,” (3) “On the Move,” (4) “Big Families,” (5) “Bonds That Tie,” (6) “Finding Your Place,” (7) “Hostile Homes,” and (8) “Stepping Up.” This docuseries was chosen based on previous research that has utilized animal nature documentaries as a passive control group that consisted of participants watching digital media and not being prompted to do or respond to anything [35,36], and lacking proper education on how to practice nonjudgmental present moment mindful awareness.

Rowing Machine and Exercise Task. An Aviron Tough Series Rower was utilized for all experimental trials. The Aviron Tough Series Rower is a full-body exercise machine with a dual air and magnetic resistance system and a 22-inch high-definition touchscreen. The resistance level was set to 1 (no resistance) for all sessions and participants. This low resistance setting was chosen to allow participants to focus more on internal sensations and attentional allocation during the task, rather than being solely driven by high physical exertion [37,38,39]. Participants were instructed to row at a self-selected pace that felt good to them. Using a low-resistance setting minimized the physiological load, allowing the study to isolate the psychological effects of the intervention from the confounding fatigue or physiological training adaptations associated with high-intensity exercise over the four-week study period [40,41]. Prior research shows that imposed intensities reduce pleasure, which can adversely impact attention and performance, supporting the choice of a self-paced design [42]. It also aimed to minimize potential injury risk and ensure consistency across all participants.

A rowing task was selected because it is a full body aerobic exercise that does not put as much stress on joints, such as the knees, compared to running. This movement involves calves, hamstrings, glutes, quadriceps, abdominals, pectorals, biceps, triceps, deltoids, and latissimus dorsi muscles.

### 2.3. Procedure

Prior to the study, Institutional Review Board (IRB) approval was obtained, and participants signed the informed consent before participation. Demographic information was obtained during visit one, and participants were excluded from continuation if they failed to meet the inclusion criteria or met any of the exclusion criteria. The study consisted of ten visits over four weeks, with visits one and ten acting as pre-assessment and post-assessment, respectively, with no video material preceding the rowing task. Visits two through eight consisted of watching an episode of the assigned Netflix series, followed by a 25 min rowing task. The mindfulness group watched an episode of *Headspace Guide to Meditation*, and the placebo group watched an episode of *Wild Babies*. Participants completed the four-week study by coming into the lab two or three times a week for a total ten visits.

Participants were instructed to row at whatever intensity they desired, and the resistance of the rower was set to no resistance. During the 25 minute (min) rowing task, RPE and attention were self-reported after a five-minute warm-up, at 12.5 min, and at 20 min before a five-minute cooldown. HR was recorded by the researcher at the same time points as discrete readings from the Polar Beat app, which received data continuously from the Polar H10 sensor. Performance outcome variables of power output, distance rowed, and strokes per minute were recorded from the rower after each rowing task by researchers. To reduce possible social facilitation effect, participants were tested individually in a private room. The participants were instructed not to share any details about the research study with others.

### 2.4. Data Analysis

After the 32 participants completed all assessments, data were analyzed using the Statistical Package for Social Sciences (SPSS) Version 29 (IBM Corp., Armonk, NY, USA). A series of 2 (Group: Mindfulness, Placebo) × 2 (Session: Pre, Post) × 3 (Time within Session: 5 min, 12.5 min, 20 min) mixed-model repeated measure analyses of variance (RM-ANOVAs) were conducted to examine changes in the primary dependent variables of RPE, HR, and attentional allocation. Separate RM-ANOVAs were conducted to test specific hypotheses for conceptually distinct domains (psychological and physiological). To control family-wise error rates given the multiple comparisons, a Bonferroni correction was applied. When Mauchly’s sphericity reached significance (*p* < 0.05), the Greenhouse–Geisser (GG) epsilon correction coefficient was implemented. Significance level for all tested measures was set at α < 0.05. A bivariate correlation assessed the relationship between RPE and HR. A series of 2 (Group: Mindfulness, Placebo) × 2 (Session: Pre, Post) factorial analyses of variance (ANOVAs) assessed performance variables of power output, distance rowed, and strokes per minute. Demographic information was analyzed with descriptive statistics to calculate means and standard deviations, along with frequencies.

## 3. Results

### 3.1. In-Task Measure Analyses

**Figure 1:**
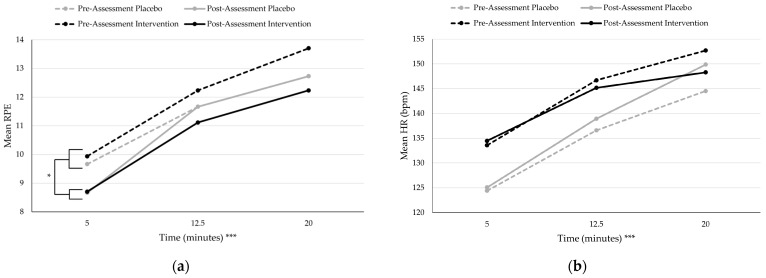
Mean Rating of Perceived Exertion (RPE) and Heart Rate (HR) over Time by Condition. * *p* < 0.05, *** *p* < 0.001. (**a**) RPE significantly increased during the rowing task, and there was a significant difference between pre-assessment RPE and post-assessment RPE regardless of group. (**b**) HR significantly increased during the rowing task, with no significant differences between session or condition.

**Figure 2:**
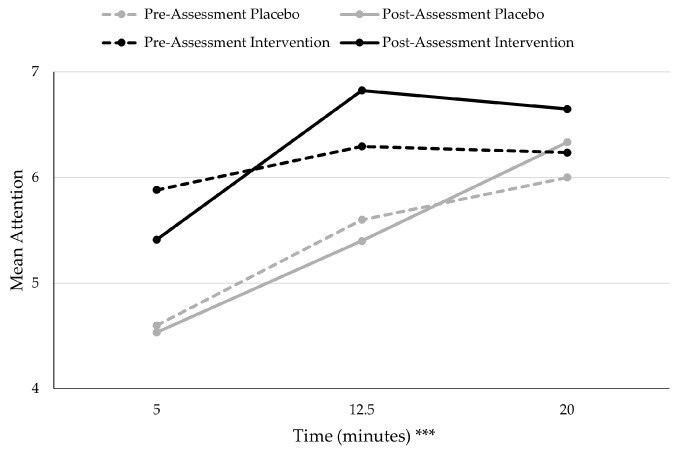
Mean Attention over Time by Condition. *** *p* < 0.001. Attention significantly increased during the rowing task, indicating more associative attention being used by participants with no significant differences between session or condition.

Crucially, the in-task measures of RPE, HR, and attention found no significant differences between the intervention and placebo group. However, there were significant differences across time for the three variables (see Table 1). RPE significantly increased linearly across time during the rowing task at the five-minute (*M* = 9.25 ± 1.89), 12.5 min (*M* = 11.67 ± 2.30), and 20 min (*M* = 12.86 ± 2.60) time points for both groups (see Figure 1). HR also significantly increased linearly across the different time points of five minutes (*M* = 129.67 ± 19.23), 12.5 min (*M* = 142.11 ± 22.66), and 20 min (*M* = 148.95 ± 24.54) (see Figure 1). Attention scores significantly increased, indicating more associative attention, from five minutes (*M* = 5.14 ± 2.46), 12.5 min (*M* = 6.06 ± 2.47), and 20 min (*M* = 6.31 ± 2.47) (see Figure 2). RPE was the only in-task measure to find a significant difference between sessions, where the RPE at the pre-assessment (*M =* 11.67 ± 2.48) was significantly greater than the RPE at the post-assessment (*M* = 10.84 ± 2.90).

### 3.2. Relationship Between the In-Task Variables of RPE and HR

When analyzing all participants, RPE and HR were found to be moderately positively correlated at all time points for both sessions (see Table 2). However, upon subgroup analysis, the placebo group demonstrated a moderate positive correlation between RPE and HR only during the post-assessment session. The intervention group demonstrated a positive correlation between RPE and HR during the pre-assessment session, and at only the 20 min time point within the post-assessment. This inversion of correlation patterns can be seen in Table 2, denoted by significance within the placebo post-assessment and intervention pre-assessment.

### 3.3. Performance Outcome Variables Analyses

#### 3.3.1. Power Output

There was not a significant main effect for group (*F* (1, 30) = 1.11, *p* = 0.30, *η_p_*^2^ = 0.04); however, there was a significant main effect for session (*F* (1, 30) = 8.20, *p* = 0.008, *η_p_*^2^ = 0.22) as the average power at the pre-assessment (*M* = 77.34 ± 39.47) was significantly less than that of the post-assessment (*M* = 87.16 ± 41.88). There was not a significant session by group interaction (*F* (1, 30) = 1.38, *p* = 0.25, *η_p_*^2^ = 0.04).

#### 3.3.2. Distance Rowed

There was not a significant main effect for group (*F* (1, 30) = 0.66, *p* = 0.42, *η_p_*^2^ = 0.02); however, there was a significant main effect for session (*F* (1, 30) = 7.06, *p* = 0.013, *η_p_*^2^ = 0.19) as the distance rowed at the pre-assessment (*M* = 4400.28 ± 685.17) was significantly less than at the post-assessment (*M* = 4558.81 ± 713.06). There was not a significant session by group interaction (*F* (1, 30) = 3.05, *p* = 0.09, *η_p_*^2^ = 0.09).

#### 3.3.3. Strokes per Minute

There were no significant main effects for group (*F* (1, 30) = 0.26, *p* = 0.61, *η_p_*^2^ = 0.009) or session (*F* (1, 30) = 0.03, *p* = 0.87, *η_p_*^2^ = 0.001). There was not a significant session by group interaction (*F* (1, 30) = 0.004, *p* = 0.95, *η_p_*^2^ = 0.000).

## 4. Discussion

The primary aim of this study was to examine the effects of a four-week, low-dose mindfulness intervention. While the results did not support the initial hypotheses regarding significant group differences in RPE, HR, attentional allocation, or performance outcomes, the study nevertheless provides insights that contribute to the growing body of literature on mindfulness and exercise. Previous research [21,22] has investigated the use of a guided meditation stimulus during the exercise task, whereas the current study innovatively considered the impact of a mindfulness meditation conducted before the exercise task. The present findings serve to inform future research that simply performing a mindfulness meditation before an exercise task may not be sufficient to observe psychophysiological differences, and further support previous studies usage of a mindfulness meditation during an exercise task [21,22].

Based on the results from this study, there were no significant differences in RPE between the two groups. This finding is not consistent with research from Cox and colleagues [21] and Solk and colleagues [22], who found that a mindfulness intervention group who utilized a guided meditation track during the exercise task experienced lower RPE than a placebo group with no stimulation. This could be due to methodological differences between the study design, that when prompted to practice mindfulness during the exercise task rather than just prior, it encourages real-time application of the skill. It also contrasts with the results of Ivanova et al. [43], who reported decreases in RPE among participants who engaged in a brief mindfulness intervention prior to listening to music during a cycling task, compared to the placebo group, which only listened to music during the exercise. In these studies, participants were exposed to an audio stimulus while performing the exercise task, whereas the present study involved participants engaging with an audiovisual stimulus prior to the rowing task, without any audio stimulus during the exercise itself [21,43].

Current literature lacks concrete findings on the relationship between mindfulness training and RPE, as there is considerable variation in the delivery of mindfulness interventions and the presence of external stimuli during exercise. Although no significant group differences were found, RPE was significantly lower at the post-assessment compared to the pre-assessment across the entire sample (*p* = 0.013, *η_p_*^2^ = 0.19). While statistically significant, this observed decrease in RPE (approximately 0.83 units on the 6–20 Borg scale) suggests a relatively small practical effect. This modest reduction in perceived exertion across all participants could potentially be attributed to increased familiarity with the rowing task over the four-week intervention period, or a general habituation to the exercise environment. This aligns with the overall sample performing better at the post-assessment by rowing at a greater distance and power, despite reporting that they were not working as hard. These findings should also be interpreted within the context of the low-intensity, self-paced task. It is plausible that the observed effects of mindfulness, specifically a lower RPE despite improved performance, are more pronounced at lower intensities, where psychological factors may be a greater performance limiter than physiological capacity [39].

Participants were asked to complete the rowing task at an intensity level that they found comfortable and enjoyable. It is important to acknowledge that because participants rowed at a self-selected pace, the physiological stimulus was not standardized across the sample, and this inter-individual variability in effort could have influenced the outcomes. While no significant group differences were found for HR, the observation that the intervention group’s HR was numerically higher at post-assessment, while their RPE was not significantly different from the placebo group, suggests a potential for altered perceived exertion that future, more robust interventions could investigate. Highlighted at the 20 min time point, the placebo group’s HR surpassed that of the intervention group, while the RPE relationship remained the same. To have reported similar perceived exertion, even though their HR during exercise was not the same, mindfulness training could have potentially influenced the participants’ perception of how hard they were exercising. This is evidenced by the lower RPE scores observed in the post-assessment compared to the pre-assessment, while HR showed no significant differences. This aligns with previous literature, where there was no difference in HR, but a reduction in RPE between a mindful exercising condition and a placebo exercising condition [22]. While the current finding aligns with previous literature [22], it is important to note methodological differences, such as Solk and colleagues’ use of a guided meditation track during the exercise task, contrasting with the present study’s pre-exercise audiovisual stimulus.

Additionally, at the post-assessment, the intervention group exhibited the highest HR among all groups, yet their RPE was the lowest across all sessions. This perception of exertion modification has been seen in previous research where mindful cycling resulted in accurate RPE prediction regarding their HR while cycling [17]. Similar findings have been reported, that when women were first taught mindfulness strategies, they reported a lower RPE and were able to tolerate exercise for longer periods of time [43].

Research historically shows a strong correlation between RPE and HR [18,19,44], which was demonstrated by the overall sample at each of the recorded time points. However, once split by group, there was no significant correlation for the placebo group at the pre-assessment, or for the intervention group at the first two time points of the post-assessment. These inconsistencies might indicate a complex interaction or inter-individual variability that the study’s design (low-dose, pre-exercise audiovisual stimulus) was not robust enough to fully capture or clarify. Mindfulness could theoretically alter the perception of exertion, leading to a decoupling of RPE and HR, even if a significant difference was not found in the overall RPE. An analysis of confidence intervals indicated wide ranges, which may lead to some uncertainty with the results; however, with minimal ranges including a zero value, it can be deduced that there was some relationship between the two variables. The incongruity between RPE and HR should not be a cause for concern in terms of understanding the primary nonsignificant group findings, but it does warrant further investigation in future research.

The absence of significant group differences for RPE, HR, attention, distance rowed, power output, and strokes per minute indicates that the intervention did not achieve its primary objectives of differentiating the mindfulness group’s responses from the placebo group. The observed inconsistencies in the RPE and HR correlation for the intervention group at the post-assessment, which deviate from established physiological principles [18,19,44], are noteworthy and warrant further investigation. While the overall lack of group effects means this inconsistency does not undermine a significant intervention finding, it highlights a complex relationship that was not fully elucidated by the current study’s design. The observation that participants, overall, reported less perceived exertion at the post-assessment while performing at a greater distance and power, with no significant difference in HR, might suggest that engagement with the intervention’s themes, even without direct real-time application, could contribute to a subtle shift in how exertion is perceived. This could potentially involve a passive coping mechanism where individuals become more familiar with the exercise and potentially more accepting of discomfort [40,45], leading to a reduced perception of exertion relative to actual work. Furthermore, this task familiarity likely involves a neuromuscular learning effect, where improved coordination and motor unit recruitment patterns lead to greater efficiency in power production, potentially contributing to the enhanced performance outcomes observed at the post-assessment [46]. However, given the lack of significant group differences, this remains a speculative interpretation that requires further research.

The results of attention allocation demonstrated no significant differences between the two groups, and no difference in average attention score for the exercise task between the pre- and post-assessment. The only significant finding within attention allocation was that attention scores became more associative as more time passed during the rowing task, similar to the increased HR as the sessions progressed. The observed increase in associative attention over time during the rowing task, consistent with the increase in HR, is a known physiological response to increasing exertion [24,40,47]. It is known that mindfulness shares properties with associative attention [21,23,27], and the intervention group at the post-assessment showed a non-significant numerical tendency towards higher associative attention at the peak of the task and sustained associative attention compared to the placebo group. However, the overall lack of significant group differences in attention allocation means a direct inference of mindfulness skill application is not statistically supported in this study. This remains an area for future investigation with more targeted real-time interventions. While also not significant, at the halfway point of the rowing task during the post-assessment, the intervention group had higher attention scores than the placebo group, indicating that they were more associative in their attention at the peak of the exercise task. The intervention group at the post-assessment also demonstrated sustained associative attention, with scores plateauing, whereas the placebo group continued to increase as the exercise progressed.

This finding of a shift towards associative attentional style is noteworthy, particularly as some literature suggests a dissociative or external focus of attention is superior for performance outcomes, especially in power- and speed-based tasks [29,30]. However, the role of attention may be task dependent. In a sustained endurance task like the one investigated in this study, an associative focus is necessary for effective pacing and effort regulation. It is proposed that the mindfulness intervention did not simply increase attention to bodily sensations through association, but rather it trained participants to appraise these sensations with nonjudgmental acceptance. This may have reframed potentially negative stimuli, such as muscle fatigue and elevated heart rate, as neutral information, preventing them from limiting performance and allowing for the observed increase in distance and power. These findings may have also been seen for the placebo group because of the calming effect of the *Wild Babies* episodes before completing the rowing task. This stimulus may have unintentionally elicited a relaxation response or mindfulness-like state, introducing a methodological confound. This possibility undermines the internal validity of the group comparisons and should be considered when interpreting the null findings. Furthermore, participants in the placebo group did not receive mindfulness instruction, indicating that they may not have known how to appropriately apply mindfulness during exercise. However, due to the active nature of the recruited sample, they may have been in tune with their body and accompanying sensations and could use associational strategies to complete the rowing task.

The significant main effect for session on both power output and distance rowed, in the absence of a group or group-by-session interaction, indicates that improvements across the four-week period were most likely driven by task familiarization rather than the specific content of the mindfulness intervention. This pattern is consistent with learning effects commonly observed in repeated-measures exercise designs, particularly when participants have limited prior experience with the modality [38,42]. All participants, regardless of group, rowed farther and generated more power at the post-assessment, suggesting that neuromuscular efficiency, improved pacing strategy, and increased comfort with the task contributed to the observed performance gains. However, it is important to note that the observed differences in distance (approximately 150 m) and power (approximately 10 watts) were associated with negligible effect sizes, suggesting limited practical significance for these overall improvements.

Although no significant effects were observed for strokes per minute, this null finding should be interpreted in the context of the task demands and the characteristics of the sample. This suggests that while participants became more efficient by rowing with more power, their rowing cadence remained stable. This may be due to the novice status of the participants, whose technical proficiency limited their ability to significantly alter stroke rate [40,45]. Overall, participants reported less perceived exertion at the post-assessment, but had no difference in their HR, and rowed at a greater distance and power. While not statistically significant, the intervention group numerically reported lower RPE despite higher HR at specific time points. Future research with larger samples is needed to determine if this pattern represents a true decoupling of perception and physiology or merely statistical noise.

The findings, despite the lack of group differences, offer practical insights for exercise professionals. The observation that participants improved performance with a lower perception of exertion after the four weeks suggests that structured, repeated exposure to an exercise task in a calming environment can be beneficial. Exercise professionals and coaches can use low-cost, technology-delivered platforms like Netflix as an accessible tool for pre-exercise relaxation or focus, which may help exercisers become more familiar with and accept, physical discomfort. This approach may be particularly promising for novice exercisers or those with psychological barriers to physical activity.

### Limitations and Future Research

This mindfulness intervention was only delivered for four weeks, whereas traditional interventions last at least eight weeks [10,48,49,50]. This intervention was also low-dose, since the participants were only watching the videos twice a week for the four weeks, and they were not encouraged to practice mindfulness on their own outside of the study. This low-dose intervention was chosen to explore the transferable effects of mindfulness teaching delivered via a readily accessible platform such as Netflix, a less intensive approach compared to a traditional eight-week intervention. Future research should increase the dosage of this intervention, using not only the episodes of *Headspace Guide to Meditation*, but also giving the participants use of the Headspace app outside of the research lab to use daily and log daily usage of the app. Researchers could also provide guidance for participants on how to apply the skills taught within the Netflix series to the exercise task to facilitate the transferability of mindfulness techniques. Future research could also implement a guided mindfulness track while the participant is exercising to allow the individual real-time application of mindfulness skills, such as guided body scan or deep breathing, alongside the educational component of *Headspace Guide to Meditation* before completing the exercise task. Additionally, future research should explore the evolution of these technological delivery platforms with the emergence of generative artificial intelligence, along with virtual and augmented reality, that could possibly amplify the effects observed in the current study.

Most of the measures were taken via self-reported verbal responses of RPE and attention during the rowing task. This could have influenced the data because the perceptions of the exercisers may be different from the reality that they were experiencing. It could also not be guaranteed that participants paid attention to the episodes of whichever show they were assigned. Future research should implement some form of biofeedback attention tracking to ensure that participants are giving the episodes their full attention, which may lead to improvements in mindfulness scores or testing the retention of the information presented. Furthermore, the exercise protocol involved a self-selected intensity, which, while ecologically valid, means it cannot be asserted that all participants experienced the same physiological stimulus. This variability is a methodological limitation and may have influenced the variance in performance outcomes. A future study implementing a standardized and higher intensity protocol would be necessary to determine if the benefits found from this study persist when physiological demand is greater.

Another limitation concerns the placebo group. Although *Wild Babies* was selected based on prior studies using nature documentaries as passive controls [48,49], the audiovisual content may have elicited a response that resembles certain affective qualities of mindfulness, such as being in nature, while lacking the key component of nonjudgmental acceptance of thoughts. Therefore, individuals in the placebo group may have unintentionally attempted to engage in mindfulness-like behaviors despite not receiving formal instruction. This represents a potential methodological confound because the placebo condition may have been effective at reducing arousal, and it may undermine the internal validity of group comparisons. Consequently, the lack of a distinct control baseline may be considered when interpreting the null findings. Future research should consider using a true control condition (e.g., waitlist control or neutral content) to avoid inadvertently inducing mindfulness-related relaxed states in the comparison group.

The study had an unequal number of males and females, with almost a four-to-one ratio of females. This was likely a result of recruitment from a population with a higher proportion of females. Since there were uneven numbers for the sexes, this impacts the generalizability of the results, and future research should investigate how males are impacted by a mindfulness intervention, and if they are as invested in using this technique in exercise. The homogenous sample of predominantly White students also reduces the generalizability of the findings. Gender socialization and cultural background play significant roles in how individuals perceive mindfulness and express physical exertion. Research indicates that traditional masculine norms emphasizing emotional control and stoicism can conflict with the mindfulness principle of nonjudgmental acceptance, potentially leading males to view acceptance as passive resignation [51,52]. Similarly, cultural mismatches between Westernized mindfulness adaptations and the values of diverse populations can create barriers to engagement [53]. Consequently, these findings may not extrapolate to male populations or those from diverse cultural backgrounds who may engage with mindfulness practices differently. To address this, future research should employ targeted recruitment strategies, such as partnering with diverse community and university organizations and utilizing stratified sampling methods, to ensure balanced representation. Doing so would enhance external validity and allow for examination of potential demographic differences and moderating factors.

## 5. Conclusions

In conclusion, while this study observed lower perceived exertion, modest shifts in attentional allocation, and improvements in distance and power output, these improvements occurred across both groups and were not specific to the mindfulness intervention. The low-dose nature of the intervention was not sufficient to elicit group differences or interaction effects, suggesting that the observed improvements were primarily due to task familiarization and repeated exposure, eliciting session effects. The study highlights that a passive, technology-delivered mindfulness intervention without real-time guidance was insufficient to elicit distinct psychophysiological benefits beyond those achieved through simple practice and repetition.

## Figures and Tables

**Table 1:** Within-between Repeated Measures ANOVAs (*n* = 32).

Dependent Variable	Main Effect	Interaction Effect	*df*	*F*	*p*	*η_p_* ^2^
Rating of Perceived Exertion (RPE)	Group		1, 30	0.04	0.84	0.001
	Session		1, 30	7.05	0.013 *	0.19
		Session × Group	1, 30	2.42	0.13	0.08
	Time		1.32, 39.52	124.25	<0.001 ***	0.81
		Time × Group	2, 60	0.12	0.89	0.004
		Session × Time	1.39, 41.61	1.15	0.31	0.04
		Session × Time × Group	2, 60	1.42	0.25	0.05
Heart Rate (HR)	Group		1, 30	1.13	0.30	0.04
	Session		1, 30	0.02	0.88	0.001
		Session × Group	1, 30	0.40	0.53	0.01
	Time		1.43, 42.74	48.35	<0.001 ***	0.62
		Time × Group	2, 60	1.26	0.29	0.04
		Session × Time	1.49, 44.71	1.46	0.90	0.00
		Session × Time × Group	2, 60	1.15	0.24	0.05
Attention	Group		1, 30	1.81	0.19	0.06
	Session		1, 30	0.05	0.83	0.002
		Session × Group	1, 30	0.03	0.87	0.001
	Time		1.41, 42.16	10.20	<0.001 ***	0.25
		Time × Group	2, 60	1.37	0.26	0.04
		Session × Time	1.44, 43.13	0.95	0.37	0.03
		Session × Time × Group	2, 60	0.72	0.49	0.02

* *p* < 0.05, *** *p* < 0.001.

**Table 2:** Bivariate correlations of Rating of Perceived Exertion (RPE) and Heart Rate (HR).

Group Assignment	Session	Time (min)	*r* [95% CI]	*p*
All Participants (*n* = 32)	Pre-Assessment	5	0.58 [0.33, 0.78]	<0.001 ***
		12.5	0.60 [0.30, 0.82]	<0.001 ***
		20	0.60 [0.28, 0.82]	<0.001 ***
	Post-Assessment	5	0.39 [0.0006, 0.73]	0.03 *
		12.5	0.44 [0.13, 0.70]	0.01 **
		20	0.63 [0.38, 0.82]	<0.001 ***
Placebo Group (*n* = 15)	Pre-Assessment	5	0.46 [−0.12, 0.86]	0.09
		12.5	0.33 [−0.23, 0.90]	0.23
		20	0.42 [−0.19, 0.91]	0.12
	Post-Assessment	5	0.55 [0.22, 0.85]	0.04 *
		12.5	0.55 [−0.12, 0.86]	0.03 *
		20	0.77 [0.28, 0.96]	<0.001 ***
Intervention Group (*n* = 17)	Pre-Assessment	5	0.67 [0.44, 0.88]	0.003 **
		12.5	0.71 [0.44, 0.89]	0.001 ***
		20	0.70 [0.35, 0.87]	0.002 **
	Post-Assessment	5	0.40 [−0.27, 0.83]	0.18
		12.5	0.43 [0.05, 0.71]	0.09
		20	0.56 [0.20, 0.81]	0.021 *

* *p* < 0.05, ** *p* < 0.01, *** *p* < 0.001.

## Data Availability

The data presented in this study are available on request from the corresponding author due to the data presented in this manuscript is a subset of a larger, ongoing research project still under active analysis.
